# Cannabis Use in Pregnant and Breastfeeding Women: Behavioral and Neurobiological Consequences

**DOI:** 10.3389/fpsyt.2020.586447

**Published:** 2020-11-02

**Authors:** Francisco Navarrete, María Salud García-Gutiérrez, Ani Gasparyan, Amaya Austrich-Olivares, Teresa Femenía, Jorge Manzanares

**Affiliations:** ^1^Instituto de Neurociencias, Universidad Miguel Hernández-CSIC, Alicante, Spain; ^2^Red Temática de Investigación Cooperativa en Salud (RETICS), Red de Trastornos Adictivos (RTA), Instituto de Salud Carlos III, MICINN and FEDER, Madrid, Spain

**Keywords:** cannabis, tetrahydrocannabinol, pregnancy, breastfeeding, mother, offspring

## Abstract

Nowadays, cannabis is the most consumed illicit drug. The global prevalence of the use of cannabis in 2017 was estimated in 188 million of people, 3.8% of worldwide population. Importantly, the legalization of cannabis in different countries, together with the increase in the apparent safety perception, may result in a great variety of health problems. Indeed, an important concern is the increase in cannabis use among pregnant and breastfeeding women, especially since the content of delta9-tetrahidrocannabinol (THC) is currently around 2-fold higher than it was 15–20 years ago. The purpose of this study was to review cannabis use during pregnancy and breastfeeding including epidemiological aspects, therapeutic or preventive strategies, and experimental considerations and results from animal models of perinatal cannabis exposure to analyze the underlying neurobiological mechanisms and to identify new therapeutic approaches. A recent report revealed that among pregnant women aged 15–44, last month cannabis use prevalence was over 4.9%, raising to 8.5% in the 18–25-year-old age range. Pre- and post-natal exposure to cannabis may be associated with critical alterations in the newborn infants that are prolonged throughout childhood and adolescence. Briefly, several reports revealed that perinatal cannabis exposure was associated with low birth weight, reduction in the head circumference, cognitive deficits (attention, learning, and memory), disturbances in emotional response leading to aggressiveness, high impulsivity, or affective disorders, and higher risk to develop a substance use disorder. Furthermore, important neurobiological alterations in different neuromodulatory and neurotransmission systems have been associated with cannabis consumption during pregnancy and lactation. In spite of the evidences pointing out the negative behavioral and neurobiological consequences of cannabis use in pregnant and breastfeeding women, there are still limitations to identify biomarkers that could help to establish preventive or therapeutic approaches. It is difficult to define the direct association specifically with cannabis, avoiding other confusing factors, co-occurrence of other drugs consumption (mainly nicotine and alcohol), lifestyle, or socioeconomic factors. Therefore, it is necessary to progress in the characterization of short- and long-term cannabis exposure-related disturbances.

## Introduction

*Cannabis sativa* contains more than 400 active chemicals and over 100 unique cannabinoids (1), the most prominent being trans-Δ-9-tetrahydrocannabinol (THC) as the main psychoactive constituent and cannabidiol (CBD) also produced in high concentrations but without abuse liability (2–5). The effects induced by cannabis use are mediated by the endocannabinoid system (ECS), mainly through two transmembrane domain and G-protein-coupled receptors (GPCRs), cannabinoid receptor type 1 (CB1r), and type 2 (CB2r).

Nowadays, various types of preparations of *C. sativa* are estimated to be consumed by 200–300 million people around the world, particularly among the young people (6, 7). It is the most popular illicit drug of the twenty-first century (United Nations Office on Drugs and Crime, UNODC) (8). Unfortunately, due to this growing demand in recreational activities, consumption trends increase rapidly and unexpectedly promoting the development of new synthesized cannabinoids substances (e.g., K2, spice) and certain modifications of the plant, especially those involving the increase in the concentration of THC to satisfy market expectations. For instance, values of THC were below 2% before 1990s; however, in 2017, there was a strain whose content was modified to reach concentrations between 17 and 28% (9). In addition, according to a recent study in Europe, the mean THC concentration was doubled between 2006 and 2016 both in the resin (from 8 to 17%) and in the grass (from 5 to 10%) of *C. sativa* plant (10). These changes cause greater potency in the negative psychoactive effects than those usually caused by cannabis itself (11).

The current legal landscape surrounding cannabis is surprisingly complex and unsettled. For example, 11 states and several municipalities of the United States (US) legalized medical cannabis (12). Furthermore, in Latin America, there are seven countries with a permissive legislation regarding the license for the use of cannabis (Chile, Peru, Mexico, Colombia, Bolivia, Argentina, and Uruguay, the latest being the first country in the world to legalize the cultivation and sale of cannabis in 2013) (13). The emergence of more permissive laws has led to the misperception of cannabis as a harmless substance, which is a major potential risk. A concerning study registered the incidence of cannabis use in children and teenagers aged 0–19 years from Massachusetts (98 calls were single substance and 120 polysubstance). The exposure cases were higher in male individuals (60.6%) than female individuals (39.4%) (14).

Certain reports show that nearly 10% of cannabis users consume this drug for medicinal purposes (15). In this regard, a series of randomized clinical trials have been developed with the purpose of investigating the short-term efficacy of smoked cannabis for neuropathic pain (16, 17), as an appetite stimulant especially for AIDS patients (18) or as an antiemetic drug in cancer chemotherapy (19). Notwithstanding the short-term efficacy for nausea, a recent approved and worrying application of medical cannabis is the alleviation of morning sickness and nausea in pregnant women (20, 21). Despite the difficulties to measure prenatal cannabis use (22), recent studies report that prevalence of cannabis use by pregnant women is increasing, and almost daily use was reported (16.2%) (23). Census divisions in the Midwest and West of US recently experienced the fastest changes among cannabis use treatment admissions of pregnant women (24). According to a study performed from 2018 to 2019, the consumption during the year before pregnancy increased daily from 1.17 to 3.05%, weekly from 1.39 to 2.73%, and monthly from 4.26 to 6.74%. Additionally, during pregnancy, daily use increased from 0.28 to 0.69%, weekly from 0.49 to 0.92%, and monthly from 1.18 to 1.77% (25).

THC and other cannabinoid compounds rapidly and efficiently cross the placenta and accumulate into the breast milk of nursing mothers (26, 27) producing multiple dose-dependent abnormalities in rodents (28). However, there are limited clinical reports evaluating the teratogenesis potential in exposed human fetuses or the neurodevelopmental alterations induced in lactating infants exposed to cannabis. Meanwhile, the mechanisms underlying the effects of cannabis on pregnancy and pregnancy outcome are poorly understood. It is important to mention that epigenetic modifications triggered by environmental factors during early life such as cannabis exposure might be related to the development of neuropsychiatric disorders in later life stages (29–32). Thus, clinical and preclinical studies are warranted to improve the knowledge regarding the potential negative consequences of perinatal cannabis use, particularly taking into consideration the actual legal and social cannabis landscape.

## Cannabis Use During Pregnancy

### Critical Involvement of the Endocannabinoid System in the Female Reproductive System and the Fetus Development

The ECS is critically involved in human fertility, and its components (enzymes, ligands, and receptors) are found in reproductive structures. Anandamide (AEA) is present in the human ovary, playing a crucial role in folliculogenesis, preovulatory follicle maturation, oocyte maturity, and ovulation (33, 34). AEA concentrations in follicular fluid appears to be correlated with oocyte quality and maturation. In this context, recent human studies indicated that plasmatic concentrations of AEA fluctuate during the menstrual cycle and the first stages of pregnancy. Clinical data suggest that high plasmatic concentrations of AEA are required for the ovulation, whereas in the period of embryo implantation and maturation, fatty acid amide hydrolase (FAAH) activity is upregulated (34). Indeed, high plasmatic AEA concentrations due to low FAAH activity in peripheral lymphocytes are predictive of spontaneous miscarriage (35, 36). Therefore, low plasmatic AEA concentrations are necessary to achieve a successful pregnancy (37). Indeed, uterine receptivity strongly depends on AEA concentrations designing the receptive area with low AEA concentrations and non-receptive area with high AEA levels (38). 2-Arachidonoylglycerol (2-AG) distribution is similar to AEA, suggesting the participation of these ligands in the early phases of the pregnancy and in the implantation regulation. This evidence was supported in studies where embryos were exposed to high levels of AEA showing embryotoxicity, reduced trophoblast implantation, and implantation failure (39–41). Similarly, women exposed to *in vitro* fertilization program and achieving a successful implantation present low AEA concentration associated with high FAAH concentrations in their peripheral lymphocytes (42). A high FAAH activity during the first trimester and low activity in the early second trimester represent a profertility factor and predicts a successful pregnancy. This idea was sustained in recent studies where low AEA plasmatic levels were detected in healthy women in the first trimester of gestation (35) but high levels in blood and placental tissues of women presenting spontaneous miscarriage (42). Here, decreased activity and expression of FAAH in maternal lymphocytes could act as an early marker for the first trimester miscarriage. Supporting these data, very low levels of FAAH were detected in placental tissues from women with spontaneous miscarriage (43).

ECS components were detected not only at the plasmatic level but also in the human reproductive structures. High levels of FAAH were found in the human cytotrophoblast and syncytiotrophoblast, suggesting its protective role modulating AEA concentrations and preventing AEA from crossing to fetus by the placenta (44, 45). FAAH and progesterone appear to show the same fluctuations during the menstrual cycle, indicating its correlation and implication as AEA concentrations modulators (46). Consequently, AEA levels during the period may be controlled by gonadotrophins, estrogen, or its combination (37). Furthermore, ECS receptors were detected in several reproductive organs and structures in different gestational phases, and its implication in achieving a successful pregnancy has been suggested. Both CB1r and CB2r were found in the medulla and cortex of the ovary and in the corpus luteum and corpus albicans (47). In addition, it was reported that ECS regulates a normal embryo transport via oviductal CB1r (48). These findings suggest that, under physiological conditions, ECS signaling through CB1r is crucial to various female reproductive events and for the normal fetal development.

In the human fetal nervous system, EC receptors play a crucial role in hardwiring the developing brain, and its distribution is different from that in adults, suggesting that endogenous and exogenous cannabinoids may present different actions in prenatal and adult organisms. ECS dynamically controls neuronal connectivity during prenatal development in the corticostriatal–thalamic circuitry and several cortical regions involved in psychiatric disorders. For instance, CB1r expression was detected in the fetal brain at 14 weeks of gestation (49), and CB1r gene expression was significantly increased in limbic structures such as in the hippocampus CA area and basal nuclear group of the amygdaloid complex at 20 weeks of gestation (50). In addition, elevated CB1r expression is present on several white neuronal tracts of the human fetus brain disappearing at the infancy (50). In contrast, in the adult human brain, CB1r gene expression is relatively prevalent in the frontal cortex, hippocampus, basal ganglia, and cerebellum (50, 51). Thus, CB1r expression changes dynamically across the gestational period in different brain regions, suggesting its crucial role in the fetal brain maturation. CB1r signaling controls long-range neuronal connectivity, and animal studies demonstrated that prenatal THC exposure induces alterations in the structure and function of cortical circuitry (52). These effects could be correlated with the alteration of CB1r-dependent regulation of both glutamatergic and GABAergic neuron development (52). In addition, AEA could be also involved in fetal brain development. AEA concentrations in the fetus brain are low at midgestation and increases gradually during postnatal development. However, 2-AG concentrations gradually increase during embryonic phase, reaching maximum concentrations immediately after birth while these normalize during postnatal development (53).

### Consequences of Cannabis Use by the Pregnant Woman on the Fetus and the Neonate

Although the pharmacokinetics of THC in adults was studied in detail, little is known during pregnancy regarding the maternal–fetal transfer of THC. Nevertheless, studies carried out in the last years indicated that after cannabis use, THC easily passes through the placenta inducing a variety of physiological effects in the fetus. THC acts as an indirect stressor to induce distress and physiological actions in later stages of life (10, 54, 55). THC molecule is highly lipophilic and is distributed rapidly to the brain and fat of the fetus after ingestion or inhalation by the pregnant mother. After maternal cannabis consumption, THC concentrations in fetal blood are approximately one-third to one-tenth of maternal concentrations. Cannabis enhances the placental barrier permeability to pharmacological and recreational substances, resulting in a potential risk factor for the fetus. The duration and magnitude of cannabis exposure and the route of administration (oral, inhalation, and different ways of smoking) are important factors involved in overall fetal toxicity (56).

Considering the distribution of ECS components in the human fetal brain, prenatal exposure to exogenous cannabinoids may modify the maturation of neurotransmitter systems and their functions through the activation of CB1r. Indeed, the binding of THC to CB1r during gestation alters the development of central dopamine and opioid neurotransmitter systems in brain areas regulating reward and motivation, increasing the vulnerability to future drug use and addiction. Postmortem studies with human fetal brains showed that prenatal THC exposure reduces dopamine D2 receptor gene expression in the basal nuclear complex of the amygdaloid system and in the nucleus accumbens. This reduction was associated with maternal cannabis consumption and was more prominent in male individuals. This fact explains, at least in part, gender differences observed in attention, learning, and memory following cannabis exposure (57). Postmortem human studies also identified that maternal cannabis use during pregnancy affects fetal expression of opioid-related genes in areas involved in emotional regulation, reward, goal-directed behavior, and motivation. Therefore, fetal exposure to cannabis might induce alterations in the limbic organization of the fetal brain, including mu-opioid and dopamine D2 receptor in several brain areas such as the amygdala or the striatum, increasing the susceptibility for the development of neuropsychiatric disorders later in life. These genetic alterations were associated with epigenetic changes. Cannabis prenatal exposure may induce alterations in epigenetic regulation of the dopamine D2 receptor gene in the nucleus accumbens, which was associated with increased heroin seeking during adulthood. Interestingly, some studies suggest that cannabis consumption in the prenatal period may induce epigenetic changes with immunological consequences for the offspring as well as long-term transgenerational effects.

Gunn et al. (58, 59) exhaustively reviewed the effect of cannabis use on a pregnant woman, as well as on neonatal parameters such as birth weight, head circumference and length, admission to the Neonatal Intensive Care Unit (NICU), gestational age, and preterm birth. They found that women who used cannabis during pregnancy presented a higher likelihood of developing anemia; however, no significant association was found with precipitated delivery (60), manual removal of the placenta (61), maternal diabetes, or premature onset of delivery (62), among many other postpartum negative outcomes (59). Children exposed to cannabis showed a decreased birth weight and a higher likelihood of needing NICU admission, whereas the statistical models employed by authors showed no association between neonatal length, head circumference, 1 and 5 min Apgar scores, gestational age, or fetal distress, among other studied variables (59). Nevertheless, this review was not able to distinguish the independent effect of cannabis since the selected population included individuals with polysubstance use. For this reason, Conner et al. (63) attempted to address this limitation evaluating specifically the effects of maternal cannabis use on neonatal outcomes by adjusting for confounding factors such as the consumption of other drugs of abuse (e.g., alcohol or tobacco). This review analyzed the relationship of cannabis use during pregnancy with some neonatal outcomes such as birth weight, preterm delivery, admission to an NICU, stillbirth, spontaneous abortion, Apgar scores, placental abruption, and perinatal death. Authors concluded that women who smoked cannabis only were not at risk for preterm delivery, but there was an association with lower mean birth weight and lower Apgar scores in neonates. However, authors pointed out that maternal cannabis use was not an independent factor given the confounding effect mainly of tobacco, which significantly increases the risk for adverse neonatal outcomes. Similarly, Varner et al. (64) showed that tetrahydrocannabinolic acid (THCA) was found in 2.9% of women with a stillbirth while in 1.7% of the controls, but according to the authors, this result may be confounded by exposure to cigarette smoking. Finally, other studies were consistent with no significant finding association between cannabis exposure during pregnancy and several negative outcomes on the mother (gestational diabetes or hypertension/preeclampsia) or the neonate (length of infant hospital stays, stillbirth, placental abruption, fetal anomalies, gestational age) (65–67).

**Figure 1:**
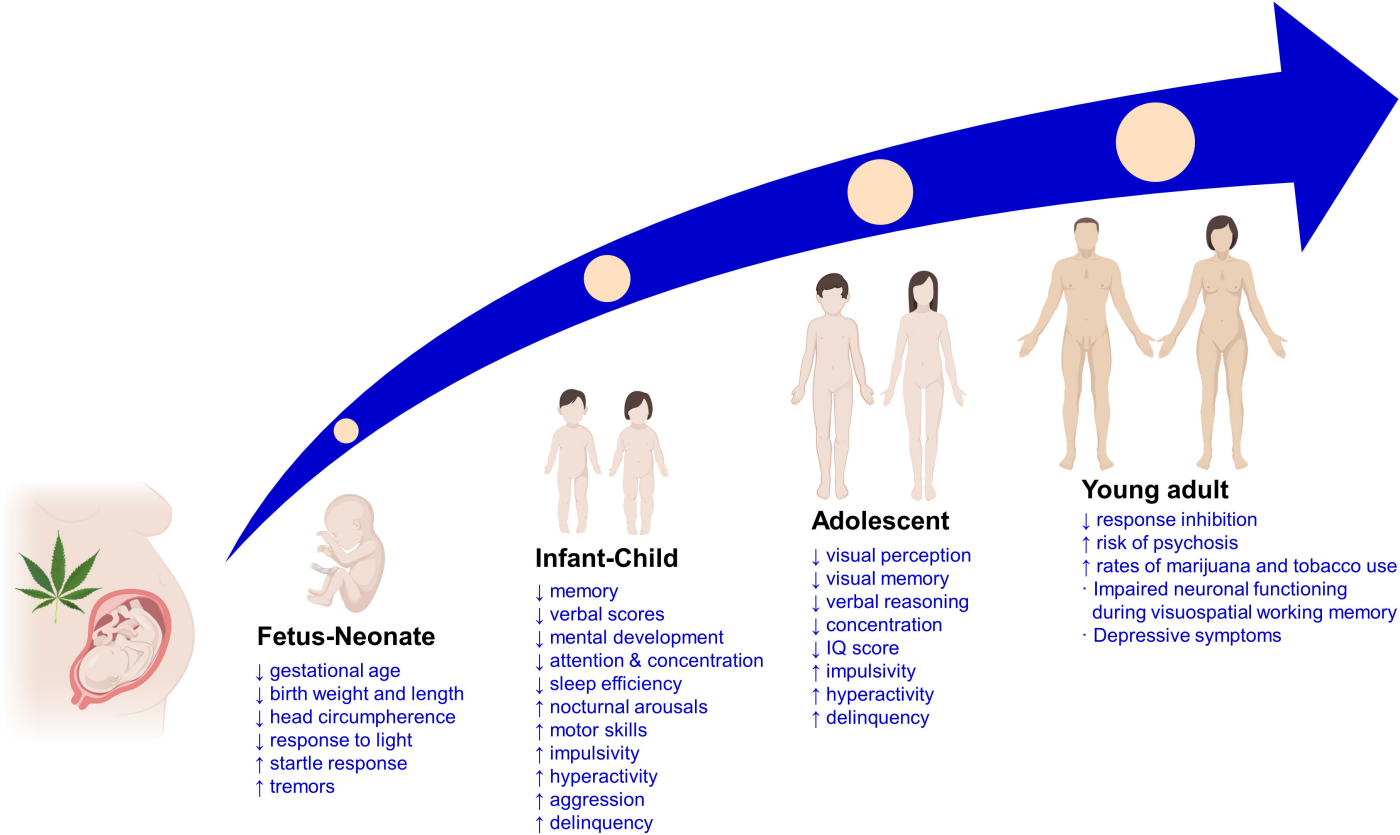
Main clinical findings of the effects of prenatal cannabis exposure on the offspring at different life stages. ↑, increased; ↓, decreased; IQ, intelligence quotient.

The effects of prenatal cannabis exposure in humans was investigated in three major prospective longitudinal clinical studies with data on the offspring beyond the early neonatal period: (i) the Ottawa Prenatal Prospective Study (OPPS) (68–71), started in 1978 with the final objective of studying the effects of cannabis used during pregnancy in white middle-class families; (ii) the Maternal Health Practices and Child Development Study (MHPCD) (72–74), started in 1982 and focused on high-risk pregnant women with low socioeconomic status, representing both white and African American women; and (iii) the Generation R study (75–81), an ongoing population-based study from the Netherlands (for more details see Table 1). All these three studies assessed the effects of cannabis exposure during the gestational period on the fetus with variability on behavioral data (82) (Figure 1).

**Table 1:** Summary of the main methodological aspects regarding the three most important longitudinal and prospective clinical studies evaluating the effect of perinatal cannabis exposure.

**STUDY**	**POPULATION**	**GOALS**	**FOLLOW-UP**	**REFERENCES**
Ottawa Prenatal Prospective Study (OPPS, started in 1978)	698 middle-class, low risk pregnant women Mostly Caucasian and predominantly Canadian cohort of women	Evaluate the effects of prenatal tobacco, alcohol, and marijuana exposure	The offspring was followed until the age of 18–22 years	(68–71)
Maternal Health Practices and Child Development Study (MHPCD, started in 1982)	564 high-risk predominantly single pregnant women with low socioeconomic status Caucasian (43%) and African American (57%) cohort of women	Evaluate the effects of prenatal alcohol and marijuana exposure	The offspring was followed until the age of 14 years	(72–74)
Generation R Study (Gen R, started in 2001**)**	9778 women living in Rotterdam (The Netherlands) Multi-ethnic cohort of women	Ongoing population-based, large-scaled study aimed to evaluate the effects of prenatal marijuana exposure on the offspring	The offspring will be followed until early childhood	(75–81)

In the neonatal population from mothers consuming cannabis during pregnancy, several physiological and behavioral alterations were observed. Researchers of the OPPS and MHPCD studies found a relationship between prenatal cannabis use and preterm births, miscarriages, pregnancy complications, low Apgar scores, and physical abnormalities in the neonates. In addition, results from the OPPS showed a decrease in the length of gestation by 0.8 weeks associated with heavy cannabis use. In contrast, MPHCD study found an increase in birth weight in neonates exposed to cannabis during the third trimester of gestation. In the Generation R study, where the fetal growth was measured by ultrasonography, an independent effect of cannabis use was found especially when cannabis use by the pregnant mother began early in pregnancy and continued throughout the entire pregnancy. Furthermore, Generation R study assessed the effect of paternal cannabis use reporting an association with fetal growth. Fetal circulation variables were also assessed in the Generation R in neonates, showing an increase in fetal pulsatility index (variability in blood velocity in a vessel). In addition, cannabis exposure during pregnancy was associated with elevated resistance index of the uterine artery, suggesting increased placental resistance. This effect could be related with reduced oxygen and nutrients accessibility, limiting a proper organogenesis that may be detrimental for the development of the fetus nervous system (82–84). Finally, a recent population-based retrospective cohort study in Ontario (Canada) was aimed to evaluate the association between self-reported prenatal cannabis use and adverse perinatal outcomes. From a cohort of 661,617 women, 9,427 (1.4%) reported cannabis use during pregnancy, and this was associated with greater frequency of preterm birth, small for gestational age, placental abruption, transfer to a NICU, and 5-min Apgar score <4 (85, 86).

### Long-Term Consequences of Prenatal Cannabis Exposure During Childhood, Adolescence, and Early Adulthood

Nowadays, the scarce clinical data regarding the long-term adverse effects of cannabis use during pregnancy on the offspring mainly come from the previously mentioned OPPS and MHPCD longitudinal studies. Apart from evaluating the consequences of the prenatal exposure to cannabis on the pregnant woman, the fetus, and the neonate, these studies also analyzed behavioral and cognitive development disturbances during childhood, adolescence, and early adulthood life stages (83, 87) (Figure 1).

#### Childhood

Initial observable effects in cannabis-exposed children were noticeable at 4 years of age in OPPS showing impaired mental development evaluated by means of response, memory, learning, vocalization, and verbal parameters (88). The MHPCD study detected impaired mental development at 9 months of age (89). However, these cognitive deficits were not reproduced in the Generation R study, but there was evidence of increased aggression and inattention levels in girls (79). In addition, disturbances in cognitive behavioral aspects regarding executive function domains, such as attention, planning, or working memory, were also described, entailing a significant impact on daily life experiences. In this respect, prenatal cannabis exposure seems to critically affect attention/impulsivity and problem-solving situations that require integration and manipulation of basic visuoperceptual skills (68). Furthermore, MHPCD study provided important information regarding intellectual abilities and school achievement, revealing that cannabis exposure during the first trimester predicted deficits in reading and spelling, as well as lower child performance, whereas cannabis use during the second trimester was associated with impaired reading comprehension (90). On the other hand, both OPPS and MHPCD studies revealed that those children exposed to cannabis during pregnancy show externalizing behavior symptoms, including hyperactivity, inattention, impulsive symptoms, and delinquency (91–93). Moreover, maternal cannabis use during pregnancy was associated with the development of psychotic-like experiences in the offspring at 10 years of age (94). Despite the evidence, in a 2017 report by the US National Academies of Sciences, the committee did not identify a good- or fair-quality systematic review that reported the association between prenatal cannabis exposure and later negative outcomes for children. This could be explained, at least in part, by the critical presence of confounding factors such as the coabuse of other drugs (i.e., tobacco, alcohol).

#### Adolescence

Despite the high variability of results during childhood when evaluating the effects of prenatal cannabis use, there is a fair described association consistency for adolescents and young adults. Data from OPPS showed reduced visual perception and increased impulsivity at 9–12 years and decreased concentration, visual memory, and verbal reasoning at 13–16 years. Moreover, the MHPCD study revealed a decrease in abstract and visual reasoning, concentration, internalization, learning and memory, and IQ scores, along with increased externalization, depression, impulsivity, hyperactivity, and delinquency (82).

#### Early Adulthood

Previously mentioned deficits in executive functions associated with prenatal consumption of cannabis seem to be long lasting since 18–22-year-old young adults showed impaired neuronal functioning during visuospatial working memory processing, measured by functional magnetic resonance imaging (fMRI) (95). Furthermore, authors of the OPPS found higher rates of depressive symptoms at 16–21 years of age (96), and the MHPCD study showed increased risk of psychosis in young adults (97). Interestingly, both studies reported higher rates of cannabis and tobacco use in the exposed cohorts at ages ranging from 14–16 to 21 years (96–98).

## Cannabis Use During Breastfeeding and Its Consequences

Despite the limited epidemiological data about the frequency of cannabis use during breastfeeding, a report from the state of Colorado (US) revealed that 7.4 and 4% of mothers younger or older than 30 years of age, respectively, were current marijuana users. From this population, 18% consumed marijuana during breastfeeding (99). Due to the growing trend of legalizing the recreational and medical use of marijuana, the proportion of lactating cannabis-using women worryingly increased in the last years. Furthermore, there is evidence that chronic consumption of cannabis by women, especially with a diagnosis of cannabis use disorder (CUD), does not decrease during lactation.

There is very scarce data regarding the pharmacokinetics of THC into human milk, as well as of other active cannabinoid compounds contained in cannabis. However, due to the 99% protein bound, liposolubility, and low molecular weight of THC, it could pass easily to breast milk. The first study reporting the presence of THC in mother's milk was published by Pérez-Reyes et al. (100), who detected milk THC concentrations in women actively smoking marijuana during breastfeeding up to 7.5 times THC plasma concentrations. Afterwards, other studies also evaluated the presence of THC in human milk providing interesting data regarding the elevated half-life of THC in milk and its clinical implications [for a recent review, see (101)]. In addition, it is also important to consider the infant exposure to THC by passive smoking (maternal or paternal) or by the mother's exhaled breath since THC was detected for 2 h after a single cannabis cigarette (102).

A major concern regarding cannabis use during breastfeeding is the availability of unclear, inconsistent, and even opposed information from clinical guidelines and health professionals. While some promote lactation for cannabis users independently of active use (103), others recommend the absolute cessation of cannabis use during lactation (104). Thus, there is a need to establish unified and evidence-based recommendations on the risk associated with cannabis use during breastfeeding.

There is very limited and variable evidence about the effects of cannabis use during lactation on infant development. The results from a study including 27 mothers reporting smoking marijuana during breastfeeding showed no differences in growth or mental and motor development, although infants were slightly shorter (105). On the other hand, another study with 68 infants exposed to cannabis during lactation revealed a slight and dose-dependent reduction in motor development without detecting differences in mental development in comparison with matched non-exposed infants (106). In addition, other reported effects of cannabis use on breastfed infants were sedation, growth delay, low tone, and poor sucking (107).

## Preventive and Therapeutic Strategies

Drug consumption during pregnancy is a major concern for mother and offspring health. Consequently, it is necessary to screen and detect the consumption of any substance of abuse among pregnant women attending prenatal units. Although its identification is still difficult, there are evidence supporting the efficacy of routine screening in clinical history or structured questionnaires in this regard (108). It is worth to mention that screening tools should be used multiple times during gestation as the patient–physician relationship progresses. Throughout the different sessions, patients are more confident with their clinician, being more open to disclose substance use problems.

In addition, toxicological screening for determining drugs and/or metabolites in maternal and neonatal biological samples is an objective and reliable approach to identify women at risk. However, in the case of cannabis, there are some limitations. Urine remains the most used sample due to its easy accessibility and the possibility of being obtained several times throughout the pregnancy. One of the main limitations of measuring THC on urine samples lies on the fact that THC can be detectable even various months after the last cannabis consumption, hampering the identification of abstinence. Meconium and umbilical cord can also be used during the second and third trimesters. However, it is not possible to identify periods of abstinence closer to delivery, apart from its limited use based on its own nature. Newborn toxicology can be used to identify families at risk of ongoing drug consumption, allowing to take actions to protect child or initiate treatment in cases of intoxication or withdrawal. If drug use disorders are not well-treated during pregnancy, the maternal difficulties handling emotions and coping with stressful situations can increase the risk of developing physiological and/or behavioral alterations in the newborn, making more difficult the postnatal adaptation of the children and the mother. Therefore, the sooner the diagnosis of cannabis abuse or dependence during pregnancy is performed, the better therapy may be planned.

**Figure 2:**
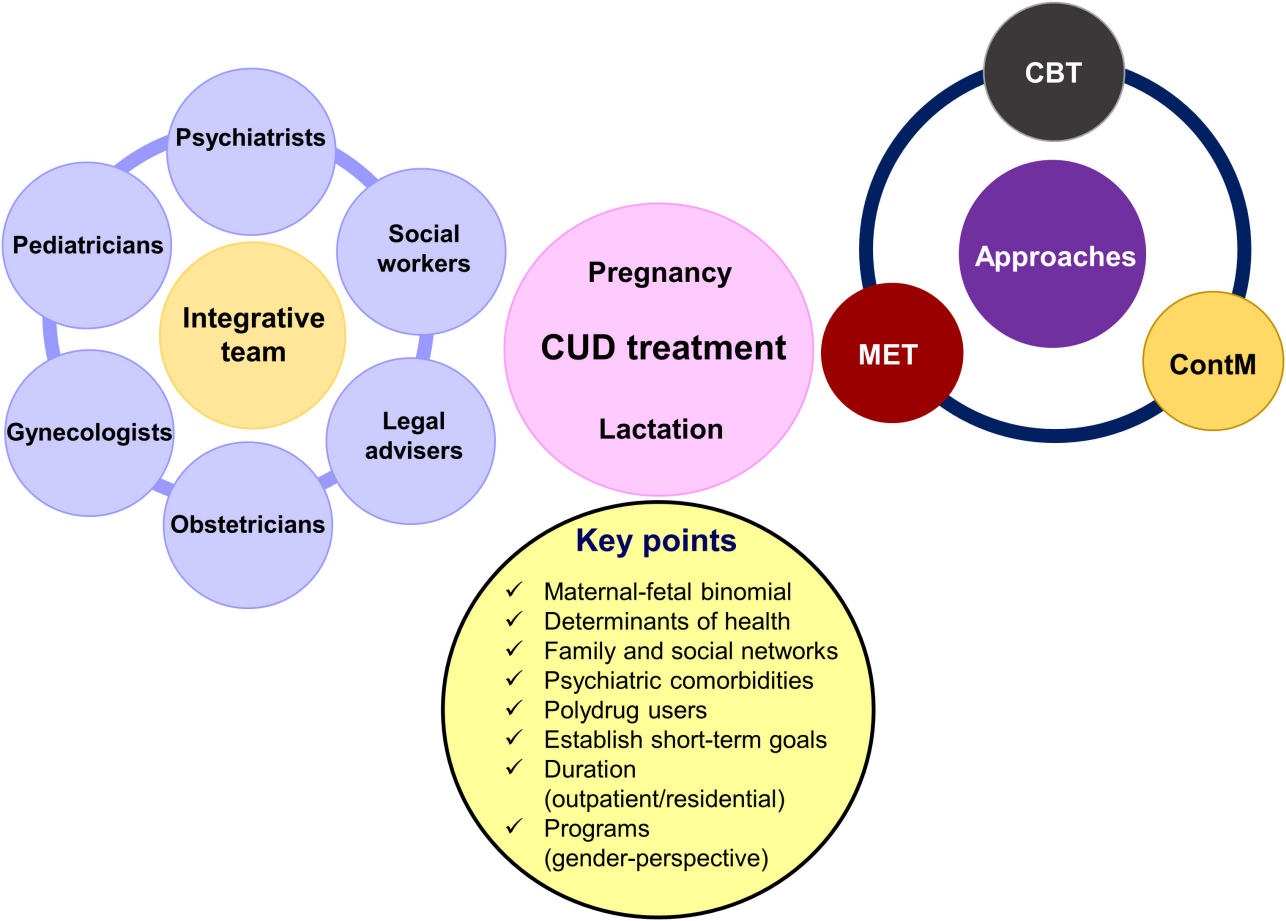
Main characteristics of comprehensive treatment for women affecting by cannabis use disorder (CUD) during pregnancy and lactation. MET, motivation enhancement treatment; CBT, cognitive–behavioral coping skills training; ContM, contingency management approach.

CUD treatment during pregnancy is integrative including a multidisciplinary team of gynecologists, obstetricians, psychiatrics, pediatricians, social workers, and legal advisers (Figure 2). The most successful treatment includes combinations of motivation enhancement treatment (MET) in association with cognitive–behavioral coping skills training (CBT) and contingency management (ContM) approaches (109, 110). It is essential to adapt treatment to the needs and peculiarities of each patient. Treatment can be outpatient or residential. Long stays in care homes are a good predictor for better abstinence rates following medical discharge, less psychiatric symptoms and legal problems, and a positive attitude to child caring (111–113). Another point to highlight is the inclusion of gender perspective in the programs (114). Specific programs designed to address the special concerns of women, including the care of their children and transportation to the treatment center, demonstrated to provide better results in comparison to traditional intensive programs. In addition, programs related to support social and familiar life improve adherence (111, 115, 116). Other strategies that provide positive outcomes are home interventions with weekly scheduled visits during the first 6 months after the delivery and then every 15 days until the year is over. Additional evidence support higher abstinence rates with home monitoring during 18 months to 3 years of duration (111, 114). The comorbidity with additional psychiatry disorders as well as polydrug use worsen patient's adherence, requiring specialized care in day hospitals or residential programs (117).

In summary, there are some key points to consider when planning treatment for women drug users during pregnancy and breastfeeding:

▪ Evaluate the main determinants of health such as access to health services and the socioeconomic level.
▪ Focus treatment on maternal–fetal binomial, bearing particularly in mind the needs of mothers to increase the motivation to achieve abstinence and not exclusively the health of the baby.
▪ Evaluate the family and social networks of each patient, with emphasis in the partner, to identify problems related with drug consumption and/or family violence.
▪ Identify comorbidities, in particular psychiatric ones.
▪ Avoid relapse during pregnancy and breastfeeding.
▪ Establish short-term goals.

Regarding breastfeeding in women with harmful use of drugs, there are for and against positions (111). The most conservative option is to discontinue lactation. Other clinicians promote continued breastfeeding except for mothers with high consumption of drugs, including cocaine, amphetamines, heroin and other opiates, benzodiazepines, or alcohol as well as in VIH+ patients. An intermediate position is to contraindicate lactation in women who consumed cannabis recently, for example in the last month previous to delivery, and to continue if patient remained abstinent during the second half of pregnancy or if she shows a clear adherence to treatment during pregnancy or postpartum. In the cases where lactation is maintained, it is advisable to make routine screening controls to stop lactation when relapse occurs.

## Animal Models of Perinatal Cannabis Exposure

Cannabis use among pregnant and lactating women could be recapitulated, at least in part, by preclinical experimental approaches in rodents. These models are fundamental to explore precisely and systematically the specific neurobiological mechanisms altered by cannabinoid compounds during brain development and the consequences on behavior and cognition.

### Neurobiological and Behavioral Alterations

In the brain, CB1r is the main target of THC and is widely expressed through many areas of the brain during development and in the adulthood. The endocannabinoid system participates in the regulation of many brain functions including neuronal proliferation, migration, morphogenesis, and synaptogenesis, as well as in regulating the mechanisms underlying several neurological and psychiatric disorders. Consequently, it is crucial to understand the long-term effects of cannabinoid exposure at this critical stage of early brain development. Current animal studies have proved important behavioral and neurochemical alterations in several brain regions of the offspring exposed to cannabis during gestation at doses considered to be equivalent to current estimates of moderate human consumption. However, the long-lasting effects of gestational cannabinoids exposure on the adult brain of the offspring are still controversial due to the low number of studies available and the use of heterogeneous designs among studies.

Cortical neurons in the adult progeny of rat dams exposed to low doses of cannabinoids during gestation show reduced long-term depression and increased excitability. In addition, gene expression changes in metabotropic glutamatergic receptor 1/5 (mGluR1/5) and transient receptor potential cation channel subfamily V member 1 (TRPV1), as well as impaired social interaction in a sex-dependent manner (118), were also described. Furthermore, THC exposure affects cortical projection neuron development of both glutamatergic and GABAergic neurons dependent of CB1r regulation leading to impaired fine motor skills, altered corticospinal connectivity, and increased seizure susceptibility (52). In the cerebellum, maternal exposure to the CB1r agonist WIN55,212-2 affected the intrinsic membrane properties of cerebellar Purkinje neurons of the offspring and decreased the rearing frequency, total distance moved, and mobility, but a significant increase in the time of righting reflex, grooming frequency, and immobility was observed. Moreover, the neuromotor function as evaluated in the grip test and balance beam test was also affected in the WIN-treated group (119). Long-lasting alterations in GABAergic hippocampal neurotransmission was present in adult rats following perinatal cannabinoid exposure (120). In addition, reduced glutamatergic neurotransmission accompanied with a decrease in astrocyte glutamate transporters (121) and impaired cortical *N*-methyl-d-aspartate (NMDA) function has also been documented (122). These alterations may account for the altered emotional reactivity (123) and memory dysfunction observed in adult rats exposed to CB1r agonists during gestation (122).

Prenatal cannabis exposure in rodents has been associated with increased vulnerability for the reinforcing and motivational actions of certain addictive substances during adolescence and adulthood. This suggests that neurodevelopmental alterations of the endocannabinoid system may affect neurotransmitter pathways associated with reward and drug dependence. Studies using rat models of perinatal THC exposure showed an enhanced morphine self-administration accompanied with changes in mu-opioid receptor binding in female brain regions related with drug reinforcement (124). Perinatal exposure to cannabinoids altered the normal development of nigrostriatal, mesolimbic, and tuberoinfundibular dopaminergic neurons in a sex-dependent and brain region restricted manner. Cannabinoid effects were marked and constant in the striatum of male subjects while alterations in limbic neurons were mostly transient, and those produced in hypothalamic neurons occurred after drug withdrawal (125, 126).

Most studies investigated the impact of *in utero* cannabis exposure in the offspring during the juvenile and adult age. However, the neurochemical changes that may occur during brain development at gestational ages are also essential to understand the concomitant mechanisms at this period and to determine the critical windows during gestation that are important for the long-term developmental outcome. Only few studies assessed the neurodevelopmental effects of cannabis in gestational brains. A study of Perez-Rosado and colleagues showed sex-dependent differences in the gene expression of the opioid peptide proenkephalin (PENK) in distinct regions of the fetal rat brain (127). Another study of Ana Bonnin et al. evaluated the gene and protein expression of the rate-limiting enzyme for dopamine synthesis, tyrosine hydroxylase (TH), and its activity in the brain of fetuses at different gestational days. Authors found increased TH gene and protein expression and activity at G14 compared to controls. Intriguingly, at G16, such effects were normalized, but the TH messenger RNA (mRNA) was again altered at GD18 and GD21 in a sex-dependent manner (128).

**Figure 3:**
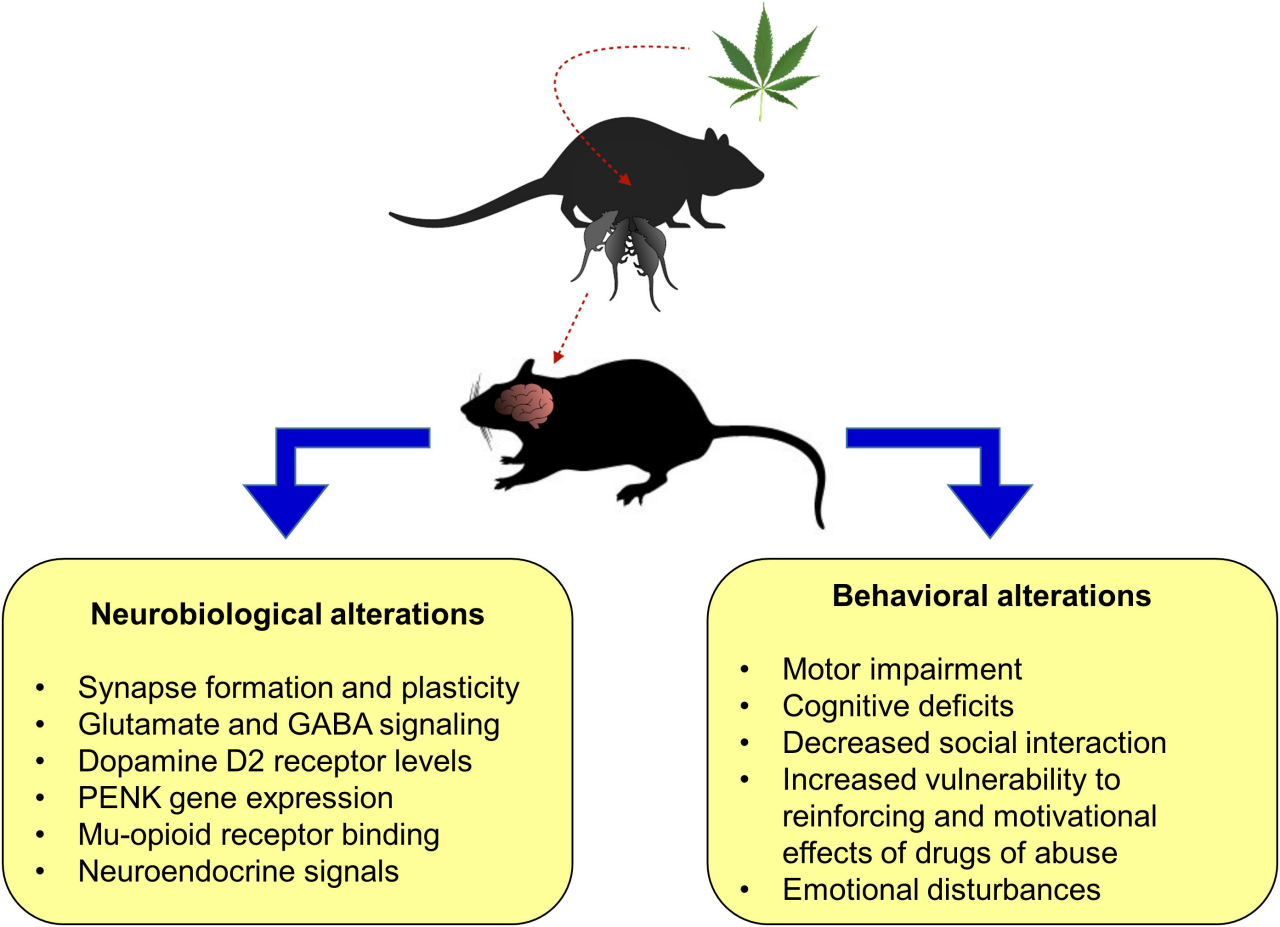
Main preclinical findings from perinatal cannabis exposure in rodent animal models on neurobiological and behavioral alterations in the litter. PENK, proenkephalin.

Animal studies play a pivotal role to provide critical clues regarding the neurobiological basis of perinatal cannabis exposure and its correlation with the clinical observations of the potential harmful effects of cannabis use during pregnancy. Indeed, preclinical studies suggest that the exposure to cannabinoids during pregnancy disrupts the normal brain development and produce long-lasting neurochemical changes. These phenomena may affect some behavioral traits later in life, increasing the susceptibility to develop neurological and neuropsychiatric disorders (Figure 3). However, the precise mechanisms require to be elucidated.

### Experimental Designs

**Figure 4:**
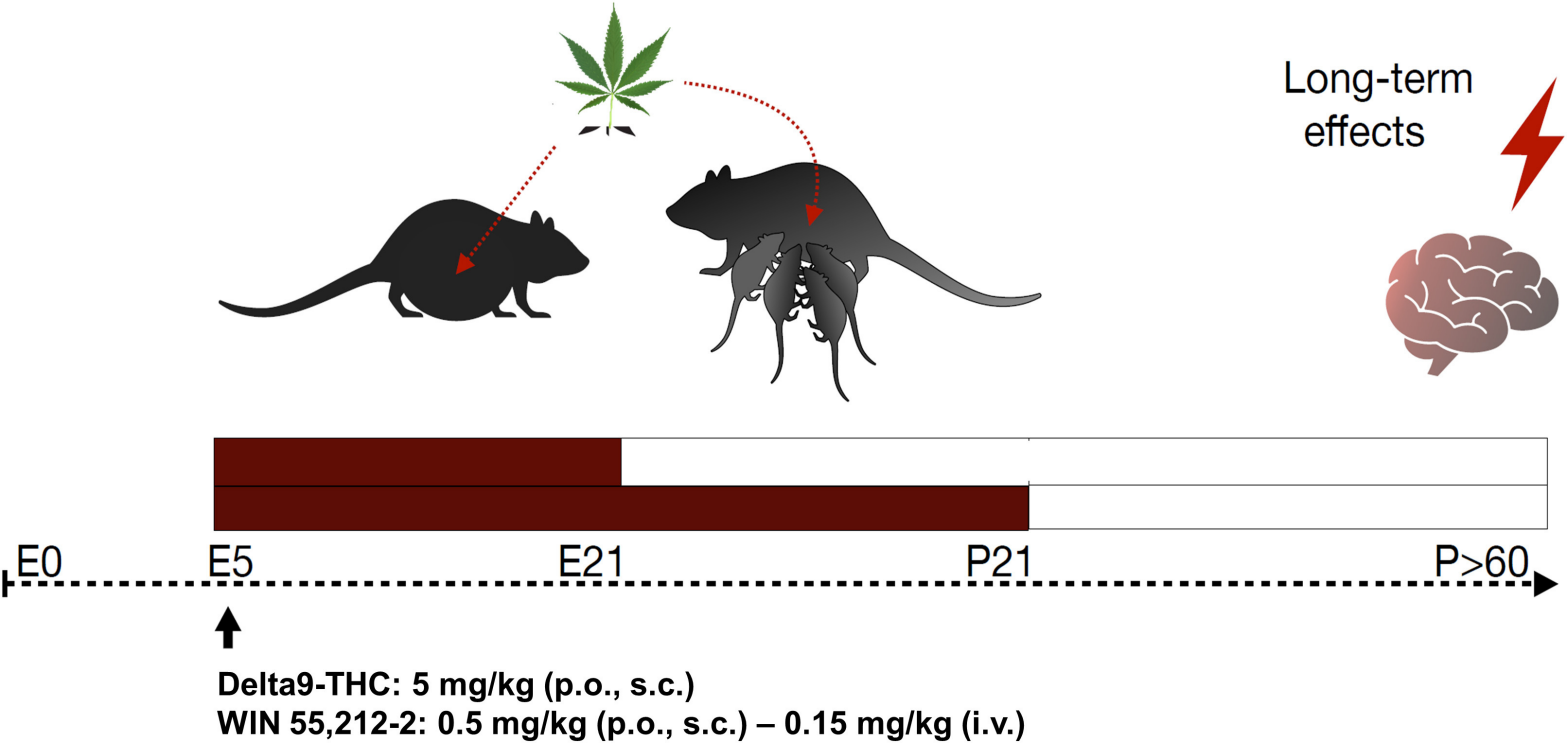
Schematic diagram of perinatal cannabis exposure animal models and long-term behavioral and neurobiological evaluation. E, embrionary; P, postnatal; Δ9THC, delta-9-tetrahydrocannabinol; p.o., per os; s.c., subcutaneous; i.v., intravenous.

This section provides an overview of currently employed perinatal cannabis exposure rodent models, attending to the main experimental aspects, and considering its potential strengths and weaknesses (Figure 4).

#### Type of Cannabis Compound

Given the chemical complexity of the cannabis plant producing over 100 phytocannabinoids as well as the novel high herbs varieties and the new synthetic cannabinoids, it is important to consider which cannabinoid compound to select. The most widely employed phytocannabinoid is THC, the major psychoactive compound of *C. sativa* plant that binds to CB1r and CB2r. In addition, the synthetic CB1r agonist WIN55,212-2 is commonly used. Some models of perinatal cannabis exposure employ crude cannabis extract, made up of several cannabinoids, including THC, cannabidiol, and cannabinol. However, the other constituents of cannabis should be taken into consideration and administered separately to precisely uncover the harmful or beneficial effects. Nevertheless, the selection of the cannabinoid compound(s) to reproduce perinatal cannabis exposure depends on the experimental question addressed by the investigator.

#### Treatment

To develop an appropriate animal model, it is important to consider the differences in the developmental ontogeny. Prenatal brain development in humans does not correspond to the same developmental period in rodents. Mouse and rat postnatal period extend up to approximately 21 days, which in humans is comparable to the third trimester of pregnancy. Usually, brain maturation among species is compared using various criteria such as cerebral growth, neurogenesis, synaptogenesis, and other variables. For instance, maximal cerebral growth speed in rodents occurs up to 8–12 postnatal days while that in humans occurs in 2–3 postnatal months. Using neurogenesis as a criterion, it has been shown that E18 and E21 rat brain match with weeks 8–9 and weeks 15–16 after fertilization in the human embryo, respectively (129). In addition, differences may occur between mice and rats. Barbara Clancy et al. developed a useful online tool that translates the specific developmental time periods across mammalian species (130). Therefore, depending on the experimental question, a good translational design to study perinatal cannabis exposure from rodents to humans needs to consider these developmental differences. Furthermore, more studies are needed to simulate the type of consumer. Some users begin the consumption during the pregnancy, to diminish anxiety or nausea, and then reduce the use during the third semester or continue it during breastfeeding. Other dams are chronic users, which might cause other types of physiological and metabolic adaptations in the body that may impact differently on the fetus.

#### Route of Administration

The selection of a proper route of administration depends on different factors including the pharmacokinetics of the drug. Smoking is the most used route of administration by pregnant women consuming cannabis. Although this route can be simulated with inhalation chambers, animal models of perinatal cannabis exposure do not employ this design. Instead, intravenous route is the one that most closely mimics the pharmacokinetics of cannabis smoking while having the advantage of rapid response, high bioavailability, and reduced irritation in response to solutions that may contain irritant diluents. However, this route presents the difficulty of an invasive surgery and the need for trained personnel. An easier and commonly employed route is through oral, but it has certain disadvantages such as poor bioavailability, first-pass effect, and the absorption can be slower or faster depending on the stomach contents (i.e., presence of food). Subcutaneous route may also be considered. Finally, some studies used the intraperitoneal administration, but this route is not advisable for pregnant female rodents.

#### Period and Dose

Most of the studies perform the treatment from gestational day 5 (GD5) and prolong it to different postnatal days (PND) depending on the gestational period to be covered from a translational point of view. For instance, some studies treat animals from GD5 to PND2, which corresponds to the human midgestation (gestation week 20), although these usually extend cannabinoid treatment until litter weaning (PND21–24). It is not adequate to start the treatment before GD5 since there is a higher risk of spontaneous abortions.

The dosage will depend on the experimental question and the type of compound used. The doses employed in animal studies are the equivalent to current estimates to moderate human exposure, and they must be corrected by the route of administration and the body surface area. Commonly used doses for THC are 1.5–5 mg/kg (p.o. or s.c.) or 0.15 mg/kg (i.v.), and for WIN55,212-2, these are 0.5–1 mg/kg (p.o. or s.c.) or 0.15 mg/kg (i.v.). However, it is worth to mention that considering the current higher THC contents in *C. sativa* plant, animal models must be updated correspondingly.

#### Litter Size

The size of the litter matters especially when studying developmental mechanisms. Following birth, if the pups continue to be studied into later developmental period, a culling of litter should be performed since litter size influences pup growth and development and a number of experimental parameters (131, 132). To mention a few, body weight gain during lactation is inversely proportional to litter size, and this is associated with milk availability. Litters more than 11 pups have shown developmental delays in maturation, such as in eye opening and pinna detachment and differences in motor behavior, reflex, emotion, and memory of the offspring. Uneven growth and development can impact on the variability of statistical analysis. The number of pups born varies depending on the strain of rat or mice used. It is desirable to keep between 8 and 10 pups, and culled litters should consist of an equal number of males and females to avoid differences in maternal behavior between both sexes.

#### Cross-Fostering Pups

Another aspect to study is the effect of the mother on the development of the pup after birth. The cross-fostering of pups after birth avoids the confounding factor of whether the developmental alterations were potentially due to a poor maternal care or abstinence behaviors of the females exposed to cannabis during pregnancy. Therefore, a safe approach is to consider cross-fostering the litter to surrogated mothers that have not undergone to any procedure. However, when cross-fostering the litter, the effects of cannabis exposure during breastfeeding disappear. This depends on the experimental question since the physiological changes that may occur in the neonatal brain could be different choosing one or another experimental paradigm.

## Concluding Remarks

Despite the limited information regarding the consequences of perinatal cannabis exposure on the offspring at different life stages, there is enough evidence to be aware of the potential risk of cannabis use during pregnancy and/or lactation. Considering the increasing rates of pregnant and breastfeeding women consuming cannabis due to the more permissive legislations of its recreational and medicinal uses, as well as the higher contents of THC in currently cannabis preparations, it is critical to establish preventive strategies to detect women at risk, especially with a CUD diagnosis, and to identify the most adequate interventions. Finally, the use of animal models of perinatal cannabis exposure is an essential tool to improve our knowledge regarding the underlying neurobiological mechanisms involved and to identify behavioral alterations avoiding the confounding factors present in clinical studies, mainly the consumption of other drugs of abuse.

## Author Contributions

FN and JM designed the sections and contents of the review manuscript. FN oversaw the organization to distribute the writing tasks among the authors and participated in manuscript writing. MSG-G, TF, AG, and AA-O perform the literature searches and participated in the manuscript writing. All authors critically reviewed and approved the final version of the manuscript.

## Conflict of Interest

The authors declare that the research was conducted in the absence of any commercial or financial relationships that could be construed as a potential conflict of interest.

## References

[1] Andre CM Hausman JF Guerriero G. *Cannabis sativa*: the plant of the thousand and one molecules. Front Plant Sci. (2016) 7:19. 10.3389/fpls.2016.00019 26870049 PMC4740396

[2] Abood ME. Allosteric modulators: a side door. J Med Chem. (2016) 59:42–3. 10.1021/acs.jmedchem.5b01824 26645411

[3] Parker LA Mechoulam R Schlievert C. Cannabidiol, a non-psychoactive component of cannabis and its synthetic dimethylheptyl homolog suppress nausea in an experimental model with rats. Neuroreport. (2002) 13:567–70. 10.1097/00001756-200204160-00006 11973447

[4] Zuardi AW Crippa JA Hallak JE Bhattacharyya S Atakan Z Martin-Santos R. A critical review of the antipsychotic effects of cannabidiol: 30 years of a translational investigation. Curr Pharm Des. (2012) 18:5131–40. 10.2174/138161212802884681 22716160

[5] Viudez-Martinez A Garcia-Gutierrez MS Medrano-Relinque J Navarron CM Navarrete F Manzanares J. Cannabidiol does not display drug abuse potential in mice behavior. Acta Pharmacol Sin. (2019) 40:358–64. 10.1038/s41401-018-0032-8 30022153 PMC6460363

[6] Sirikantaramas S Taura F Morimoto S Shoyama Y. Recent advances in *Cannabis sativa* research: biosynthetic studies and its potential in biotechnology. Curr Pharm Biotechnol. (2007) 8:237–43. 10.2174/138920107781387456 17691992

[7] Hall W Degenhardt L. Prevalence and correlates of cannabis use in developed and developing countries. Curr Opin Psychiatry. (2007) 20:393–7. 10.1097/YCO.0b013e32812144cc 17551355

[8] Cohen K Weinstein A. The effects of cannabinoids on executive functions: evidence from cannabis and synthetic cannabinoids-a systematic review. Brain Sci. (2018) 8:40. 10.3390/brainsci8030040 29495540 PMC5870358

[9] Stuyt E. The problem with the current high potency THC marijuana from the perspective of an addiction psychiatrist. Mo Med. (2018) 115:482–6. 30643324 PMC6312155

[10] Freeman TP Groshkova T Cunningham A Sedefov R Griffiths P Lynskey MT. Increasing potency and price of cannabis in Europe, 2006-16. Addiction. (2019) 114:1015–23. 10.1111/add.14525 30597667 PMC6590252

[11] CDC Centers for Disease Control Prevention - Health Studies - Understanding Chemical Radiation Exposures / Synthetic Cannabinoids. Atlanta: CDC (Centers for Disease Control and Prevention), U.S. Department of Health & human Services (2019). Available online at: https://www.cdc.gov/nceh/hsb/chemicals/sc/default.html

[12] Goncalves J Rosado T Soares S Simao AY Caramelo D Luis A. Cannabis and its secondary metabolites: their use as therapeutic drugs, toxicological aspects, analytical determination. Medicines. (2019) 6:31. 10.3390/medicines6010031 30813390 PMC6473697

[13] Caceres Guido P Riva N Calle G Dell'Orso M Gatto M Sberna N. Medicinal cannabis in Latin America: history, current state of regulation, and the role of the pharmacist in a new clinical experience with cannabidiol oil. J Am Pharm Assoc 2003. (2020) 60:212–5. 10.1016/j.japh.2019.09.012 31706799

[14] Whitehill JM Harrington C Lang CJ Chary M Bhutta WA Burns MM. Incidence of pediatric cannabis exposure among children and teenagers aged 0 to 19 years before and after medical marijuana legalization in massachusetts. JAMA Netw Open. (2019) 2:e199456. 10.1001/jamanetworkopen.2019.9456 31418807 PMC6704738

[15] Compton WM Han B Hughes A Jones CM Blanco C. Use of marijuana for medical purposes among adults in the United States. JAMA. (2017) 317:209–11. 10.1001/jama.2016.18900 27992636 PMC7391484

[16] Bowen LL McRae-Clark AL. Therapeutic benefit of smoked cannabis in randomized placebo-controlled studies. Pharmacotherapy. (2018) 38:80–5. 10.1002/phar.2064 29178487 PMC5766362

[17] Deshpande A Mailis-Gagnon A Zoheiry N Lakha SF. Efficacy and adverse effects of medical marijuana for chronic noncancer pain: Systematic review of randomized controlled trials. Can Fam Physician. (2015) 61:e372–81. 26505059 PMC4541447

[18] Bayer RE Therapeutic cannabis (marijuana) as an antiemetic and appetite stimulant in persons with Acquired Immunodeficiency Syndrome (AIDS). J. Cannabis Ther. (2001) 1:5–16. 10.1300/J175v01n03_02

[19] Brown D Watson M Schloss J. Pharmacological evidence of medicinal cannabis in oncology: a systematic review. Support Care Cancer. (2019) 27:3195–207. 10.1007/s00520-019-04774-5 31062109

[20] Young-Wolff KC Sarovar V Tucker LY Avalos LA Alexeeff S Conway A. Trends in marijuana use among pregnant women with and without nausea and vomiting in pregnancy, 2009-2016. Drug Alcohol Depend. (2019) 196:66–70. 10.1016/j.drugalcdep.2018.12.009 30711893 PMC6392046

[21] Volkow ND Compton WM Wargo EM. The risks of marijuana use during pregnancy. JAMA. (2017) 317:129–30. 10.1001/jama.2016.18612 27992628

[22] Corsi DJ. Epidemiological challenges to measuring prenatal cannabis use and its potential harms. BJOG. (2020) 127:17. 10.1111/1471-0528.15985 31610083

[23] Ko JY Farr SL Tong VT Creanga AA Callaghan WM. Prevalence and patterns of marijuana use among pregnant and nonpregnant women of reproductive age. Am J Obstet Gynecol. (2015) 213:201.e1–e10. 10.1016/j.ajog.2015.03.021 25772211 PMC7469257

[24] Martin CE Longinaker N Mark K Chisolm MS Terplan M. Recent trends in treatment admissions for marijuana use during pregnancy. J Addict Med. (2015) 9:99–104. 10.1097/ADM.0000000000000095 25525944

[25] Young-Wolff KC Sarovar V Tucker LY Conway A Alexeeff S Weisner C. Self-reported daily, weekly, and monthly cannabis use among women before and during pregnancy. JAMA Netw Open. (2019) 2:e196471. 10.1001/jamanetworkopen.2019.6471 31322686 PMC6646980

[26] Bernick SJ Kane S. Drug transfer to the fetus and to the breastfeeding infant: what do we know? Curr Drug Deliv. (2012) 9:350–5. 10.2174/156720112801323116 22762278

[27] Fantasia HC. Pharmacologic implications of marijuana use during pregnancy. Nurs Womens Health. (2017) 21:217–23. 10.1016/j.nwh.2017.04.002 28599743

[28] Gilbert MT Sulik KK Fish EW Baker LK Dehart DB Parnell SE. Dose-dependent teratogenicity of the synthetic cannabinoid CP-55,940 in mice. Neurotoxicol Teratol. (2016) 58:15–22. 10.1016/j.ntt.2015.12.004 26708672 PMC4912947

[29] Acharya KS Schrott R Grenier C Huang Z Holloway Z Hawkey A. Epigenetic alterations in cytochrome P450 oxidoreductase (Por) in sperm of rats exposed to tetrahydrocannabinol (THC). Sci Rep. (2020) 10:12251. 10.1038/s41598-020-69204-7 32704063 PMC7378842

[30] Szutorisz H Hurd YL. Epigenetic effects of cannabis exposure. Biol Psychiatry. (2016) 79:586–94. 10.1016/j.biopsych.2015.09.014 26546076 PMC4789113

[31] Szutorisz H Hurd YL. High times for cannabis: epigenetic imprint and its legacy on brain and behavior. Neurosci Biobehav Rev. (2018) 85:93–101. 10.1016/j.neubiorev.2017.05.011 28506926 PMC5682234

[32] Osborne AJ Pearson JF Noble AJ Gemmell NJ Horwood LJ Boden JM. Genome-wide DNA methylation analysis of heavy cannabis exposure in a New Zealand longitudinal cohort. Transl Psychiatry. (2020) 10:114. 10.1038/s41398-020-0800-3 32321915 PMC7176736

[33] Schuel H Burkman LJ Lippes J Crickard K Forester E Piomelli D. N-Acylethanolamines in human reproductive fluids. Chem Phys Lipids. (2002) 121:211–27. 10.1016/S0009-3084(02)00158-5 12505702

[34] El-Talatini MR Taylor AH Konje JC. Fluctuation in anandamide levels from ovulation to early pregnancy in in-vitro fertilization-embryo transfer women, and its hormonal regulation. Hum Reprod. (2009) 24:1989–98. 10.1093/humrep/dep065 19363040

[35] Maccarrone M Valensise H Bari M Lazzarin N Romanini C Finazzi-Agro A. Relation between decreased anandamide hydrolase concentrations in human lymphocytes and miscarriage. Lancet. (2000) 355:1326–9. 10.1016/S0140-6736(00)02115-2 10776746

[36] Habayeb OM Taylor AH Finney M Evans MD Konje JC. Plasma anandamide concentration and pregnancy outcome in women with threatened miscarriage. JAMA. (2008) 299:1135–6. 10.1001/jama.299.10.1135 18334688

[37] Bari M Battista N Pirazzi V Maccarrone M. The manifold actions of endocannabinoids on female and male reproductive events. Front Biosci. (2011) 16:498–516. 10.2741/3701 21196184

[38] Wang H Xie H Sun X Kingsley PJ Marnett LJ Cravatt BF. Differential regulation of endocannabinoid synthesis and degradation in the uterus during embryo implantation. Prostaglandins Other Lipid Mediat. (2007) 83:62–74. 10.1016/j.prostaglandins.2006.09.009 17259073 PMC1805469

[39] Paria BC Dey SK. Ligand-receptor signaling with endocannabinoids in preimplantation embryo development and implantation. Chem Phys Lipids. (2000) 108:211–20. 10.1016/S0009-3084(00)00197-3 11106792

[40] Yang ZM Paria BC Dey SK. Activation of brain-type cannabinoid receptors interferes with preimplantation mouse embryo development. Biol Reprod. (1996) 55:756–61. 10.1095/biolreprod55.4.756 8879486

[41] Wang H Dey SK. Lipid signaling in embryo implantation. Prostaglandins Other Lipid Mediat. (2005) 77:84–102. 10.1016/j.prostaglandins.2004.09.013 16099394

[42] Maccarrone M Bisogno T Valensise H Lazzarin N Fezza F Manna C. Low fatty acid amide hydrolase and high anandamide levels are associated with failure to achieve an ongoing pregnancy after IVF and embryo transfer. Mol Hum Reprod. (2002) 8:188–95. 10.1093/molehr/8.2.188 11818522

[43] Trabucco E Acone G Marenna A Pierantoni R Cacciola G Chioccarelli T. Endocannabinoid system in first trimester placenta: low FAAH and high CB1 expression characterize spontaneous miscarriage. Placenta. (2009) 30:516–22. 10.1016/j.placenta.2009.03.015 19419760

[44] Habayeb OM Taylor AH Evans MD Cooke MS Taylor DJ Bell SC. Plasma levels of the endocannabinoid anandamide in women–a potential role in pregnancy maintenance and labor? J Clin Endocrinol Metab. (2004) 89:5482–7. 10.1210/jc.2004-0681 15531501

[45] Helliwell RJ Chamley LW Blake-Palmer K Mitchell MD Wu J Kearn CS. Characterization of the endocannabinoid system in early human pregnancy. J Clin Endocrinol Metab. (2004) 89:5168–74. 10.1210/jc.2004-0388 15472222

[46] Lazzarin N Valensise H Bari M Ubaldi F Battista N Finazzi-Agro A. Fluctuations of fatty acid amide hydrolase and anandamide levels during the human ovulatory cycle. Gynecol Endocrinol. (2004) 18:212–8. 10.1080/09513590410001692492 15293893

[47] El-Talatini MR Taylor AH Elson JC Brown L Davidson AC Konje JC. Localisation and function of the endocannabinoid system in the human ovary. PLoS ONE. (2009) 4:e4579. 10.1371/journal.pone.0004579 19238202 PMC2640464

[48] Sun X Dey SK. Aspects of endocannabinoid signaling in periimplantation biology. Mol Cell Endocrinol. (2008) 286:S3–11. 10.1016/j.mce.2008.01.002 18294762 PMC2435201

[49] Biegon A Kerman IA. Autoradiographic study of pre- and postnatal distribution of cannabinoid receptors in human brain. Neuroimage. (2001) 14:1463–8. 10.1006/nimg.2001.0939 11707102

[50] Mato S Del Olmo E Pazos A. Ontogenetic development of cannabinoid receptor expression and signal transduction functionality in the human brain. Eur J Neurosci. (2003) 17:1747–54. 10.1046/j.1460-9568.2003.02599.x 12752773

[51] Wang X Dow-Edwards D Keller E Hurd YL. Preferential limbic expression of the cannabinoid receptor mRNA in the human fetal brain. Neuroscience. (2003) 118:681–94. 10.1016/S0306-4522(03)00020-4 12710976

[52] de Salas-Quiroga Diaz-Alonso J Garcia-Rincon D Remmers F Vega D Gomez-Canas M. Prenatal exposure to cannabinoids evokes long-lasting functional alterations by targeting CB1 receptors on developing cortical neurons. Proc Natl Acad Sci USA. (2015) 112:13693–8. 10.1073/pnas.1514962112 26460022 PMC4640742

[53] Friedrich J Khatib D Parsa K Santopietro A Gallicano GI. The grass isn't always greener: the effects of cannabis on embryological development. BMC Pharmacol Toxicol. (2016) 17:45. 10.1186/s40360-016-0085-6 27680736 PMC5041313

[54] Tawfik GM Hashan MR Abdelaal A Tieu TM Huy NT. A commentary on the medicinal use of marijuana. Trop Med Health. (2019) 47:35. 10.1186/s41182-019-0161-x 31148941 PMC6534865

[55] Cohen K Weizman A Weinstein A. Positive and negative effects of cannabis and cannabinoids on health. Clin Pharmacol Ther. (2019) 105:1139–1147. 10.1002/cpt.1381 30703255

[56] Ebbert JO Scharf EL Hurt RT Medical cannabis. Mayo Clin Proc. (2018) 93:1842–7. 10.1016/j.mayocp.2018.09.005 30522595

[57] DiNieri JA Wang X Szutorisz H Spano SM Kaur J Casaccia P. Maternal cannabis use alters ventral striatal dopamine D2 gene regulation in the offspring. Biol Psychiatry. (2011) 70:763–9. 10.1016/j.biopsych.2011.06.027 21820648 PMC3186868

[58] Gunn JK Rosales CB Center KE Nunez AV Gibson SJ Ehiri JE. The effects of prenatal cannabis exposure on fetal development and pregnancy outcomes: a protocol. BMJ Open. (2015) 5:e007227. 10.1136/bmjopen-2014-007227 25770234 PMC4360840

[59] Gunn JK Rosales CB Center KE Nunez A Gibson SJ Christ C. Prenatal exposure to cannabis and maternal and child health outcomes: a systematic review and meta-analysis. BMJ Open. (2016) 6:e009986. 10.1136/bmjopen-2015-009986 27048634 PMC4823436

[60] Greenland S Richwald GA Honda GD. The effects of marijuana use during pregnancy. II. a study in a low-risk home-delivery population. Drug Alcohol Depend. (1983) 11:359–66. 10.1016/0376-8716(83)90026-1 6617473

[61] Greenland S Staisch KJ Brown N Gross SJ. Effects of marijuana on human pregnancy, labor, and delivery. Neurobehav Toxicol Teratol. (1982) 4:447–50. 10.1016/0002-9378(82)90082-5 6981779

[62] de Moraes Barros MC Guinsburg R de Araujo Peres C Mitsuhiro S Chalem E Laranjeira RR. Exposure to marijuana during pregnancy alters neurobehavior in the early neonatal period. J Pediatr. (2006) 149:781–7. 10.1016/j.jpeds.2006.08.046 17137892

[63] Conner SN Bedell V Lipsey K Macones GA Cahill AG Tuuli MG. Maternal marijuana use and adverse neonatal outcomes: a systematic review and meta-analysis. Obstet Gynecol. (2016) 128:713–23. 10.1097/AOG.0000000000001649 27607879

[64] Varner MW Silver RM Rowland Hogue CJ Willinger M Parker CB Thorsten VR. Association between stillbirth and illicit drug use and smoking during pregnancy. Obstet Gynecol. (2014) 123:113–25. 10.1097/AOG.0000000000000052 24463671 PMC3931517

[65] Warshak CR Regan J Moore B Magner K Kritzer S Van Hook J. Association between marijuana use and adverse obstetrical and neonatal outcomes. J Perinatol. (2015) 35:991–5. 10.1038/jp.2015.120 26401751

[66] Budde MP De Lange TE Dekker GA Chan A Nguyen AM. Risk factors for placental abruption in a socio-economically disadvantaged region. J Matern Fetal Neonatal Med. (2007) 20:687–93. 10.1080/14767050701482738 17701669

[67] Leemaqz SY Dekker GA McCowan LM Kenny LC Myers JE Simpson NA. Maternal marijuana use has independent effects on risk for spontaneous preterm birth but not other common late pregnancy complications. Reprod Toxicol. (2016) 62:77–86. 10.1016/j.reprotox.2016.04.021 27142189

[68] Fried PA Watkinson B Gray R. Differential effects on cognitive functioning in 9- to 12-year olds prenatally exposed to cigarettes and marihuana. Neurotoxicol Teratol. (1998) 20:293–306. 10.1016/S0892-0362(97)00091-3 9638687

[69] Fried PA. The Ottawa Prenatal Prospective Study (OPPS): methodological issues and findings–it's easy to throw the baby out with the bath water. Life Sci. (1995) 56:2159–68. 10.1016/0024-3205(95)00203-I 7539879

[70] Fried PA. Marihuana use by pregnant women: neurobehavioral effects in neonates. Drug Alcohol Depend. (1980) 6:415–24. 10.1016/0376-8716(80)90023-X 7472153

[71] Fried PA Watkinson B. 12- and 24-month neurobehavioural follow-up of children prenatally exposed to marihuana, cigarettes and alcohol. Neurotoxicol Teratol. (1988) 10:305–13. 10.1016/0892-0362(88)90032-3 3226373

[72] Day NL Richardson GA Geva D Robles N. Alcohol, marijuana, and tobacco: effects of prenatal exposure on offspring growth and morphology at age six. Alcohol Clin Exp Res. (1994) 18:786–94. 10.1111/j.1530-0277.1994.tb00041.x 7526725

[73] Day N Sambamoorthi U Taylor P Richardson G Robles N Jhon Y Prenatal marijuana use and neonatal outcome. Neurotoxicol Teratol. (1991) 13:329–34. 10.1016/0892-0362(91)90079-C 1886543

[74] Day NL Richardson GA. Prenatal marijuana use: epidemiology, methodologic issues, infant outcome. Clin Perinatol. (1991) 18:77–91. 10.1016/S0095-5108(18)30535-9 2040119

[75] El Marroun H Tiemeier H Steegers EA Jaddoe VW Hofman A Verhulst FC. Intrauterine cannabis exposure affects fetal growth trajectories: the generation R study. J Am Acad Child Adolesc Psychiatry. (2009) 48:1173–81. 10.1097/CHI.0b013e3181bfa8ee 19858757

[76] El Marroun H Hudziak JJ Tiemeier H Creemers H Steegers EA Jaddoe VW. Intrauterine cannabis exposure leads to more aggressive behavior and attention problems in 18-month-old girls. Drug Alcohol Depend. (2011) 118:470–4. 10.1016/j.drugalcdep.2011.03.004 21470799

[77] Kooijman MN Kruithof CJ van Duijn CM Duijts L Franco OH van IMH. The generation R study: design and cohort update 2017. Eur J Epidemiol. (2016) 31:1243–64. 10.1007/s10654-016-0224-9 28070760 PMC5233749

[78] Kooijman MN Gaillard R Reiss I Hofman A Steegers E Jaddoe V. Influence of fetal blood flow redistribution on fetal and childhood growth and fat distribution: the generation R STUDY. BJOG. (2016) 123:2104–12. 10.1111/1471-0528.13933 26936012 PMC5427172

[79] Hofman A Jaddoe VW Mackenbach JP Moll HA Snijders RF Steegers EA. Growth, development and health from early fetal life until young adulthood: the generation R study. Paediatr Perinat Epidemiol. (2004) 18:61–72. 10.1111/j.1365-3016.2003.00521.x 14738548

[80] Jaddoe VW van Duijn CM Franco OH van der Heijden AJ van Iizendoorn MH de Jongste JC. The Generation R Study: design and cohort update 2012. Eur J Epidemiol. (2012) 27:739–56. 10.1007/s10654-012-9735-1 23086283

[81] Jaddoe VW van Duijn CM van der Heijden AJ Mackenbach JP Moll HA Steegers EA. The generation R Study: design and cohort update 2010. Eur J Epidemiol. (2010) 25:823–41. 10.1007/s10654-010-9516-7 20967563 PMC2991548

[82] McLemore GL Richardson KA. Data from three prospective longitudinal human cohorts of prenatal marijuana exposure and offspring outcomes from the fetal period through young adulthood. Data Brief. (2016) 9:753–7. 10.1016/j.dib.2016.10.005 27833935 PMC5096595

[83] Calvigioni D Hurd YL Harkany T Keimpema E. Neuronal substrates and functional consequences of prenatal cannabis exposure. Eur Child Adolesc Psychiatry. (2014) 23:931–41. 10.1007/s00787-014-0550-y 24793873 PMC4459494

[84] Campolongo P Trezza V Palmery M Trabace L Cuomo V. Developmental exposure to cannabinoids causes subtle and enduring neurofunctional alterations. Int Rev Neurobiol. (2009) 85:117–33. 10.1016/S0074-7742(09)85009-5 19607965

[85] Corsi DJ Hsu H Weiss D Fell DB Walker M. Trends and correlates of cannabis use in pregnancy: a population-based study in Ontario, Canada from 2012 to 2017. Can J Public Health. (2019) 110:76–84. 10.17269/s41997-018-0148-0 30387034 PMC6335373

[86] Corsi DJ Walsh L Weiss D Hsu H El-Chaar D Hawken S. Association between self-reported prenatal cannabis use and maternal, perinatal, neonatal outcomes. JAMA. (2019) 322:145–52. 10.1001/jama.2019.8734 31211826 PMC6582262

[87] Ryan SA Ammerman SD O'Connor ME. Marijuana use during pregnancy and breastfeeding: implications for neonatal and childhood outcomes. Pediatrics. (2018) 142:e20181889. 10.1542/peds.2018-1889 30150209

[88] Fried PA Watkinson B. 36- and 48-month neurobehavioral follow-up of children prenatally exposed to marijuana, cigarettes, and alcohol. J Dev Behav Pediatr. (1990) 11:49–58. 10.1097/00004703-199004000-00003 2324288

[89] Richardson GA Day NL Goldschmidt L. Prenatal alcohol, marijuana, and tobacco use: infant mental and motor development. Neurotoxicol Teratol. (1995) 17:479–87. 10.1016/0892-0362(95)00006-D 7565494

[90] Goldschmidt L Richardson GA Cornelius MD Day NL. Prenatal marijuana and alcohol exposure and academic achievement at age 10. Neurotoxicol Teratol. (2004) 26:521–32. 10.1016/j.ntt.2004.04.003 15203174

[91] Goldschmidt L Day NL Richardson GA. Effects of prenatal marijuana exposure on child behavior problems at age 10. Neurotoxicol Teratol. (2000) 22:325–36. 10.1016/S0892-0362(00)00066-0 10840176

[92] Fried PA Watkinson B Gray R. A follow-up study of attentional behavior in 6-year-old children exposed prenatally to marihuana, cigarettes, and alcohol. Neurotoxicol Teratol. (1992) 14:299–311. 10.1016/0892-0362(92)90036-A 1454038

[93] Leech SL Richardson GA Goldschmidt L Day NL. Prenatal substance exposure: effects on attention and impulsivity of 6-year-olds. Neurotoxicol Teratol. (1999) 21:109–18. 10.1016/S0892-0362(98)00042-7 10192271

[94] Bolhuis K Kushner SA Yalniz S Hillegers MHJ Jaddoe VWV Tiemeier H. Maternal and paternal cannabis use during pregnancy and the risk of psychotic-like experiences in the offspring. Schizophr Res. (2018) 202:322–7. 10.1016/j.schres.2018.06.067 29983267

[95] Smith AM Fried PA Hogan MJ Cameron I. Effects of prenatal marijuana on visuospatial working memory: an fMRI study in young adults. Neurotoxicol Teratol. (2006) 28:286–95. 10.1016/j.ntt.2005.12.008 16473495

[96] Porath AJ Fried PA. Effects of prenatal cigarette and marijuana exposure on drug use among offspring. Neurotoxicol Teratol. (2005) 27:267–77. 10.1016/j.ntt.2004.12.003 15734278

[97] Day NL Goldschmidt L Day R Larkby C Richardson GA. Prenatal marijuana exposure, age of marijuana initiation, and the development of psychotic symptoms in young adults. Psychol Med. (2015) 45:1779–87. 10.1017/S0033291714002906 25534593 PMC8128137

[98] Day NL Goldschmidt L Thomas CA. Prenatal marijuana exposure contributes to the prediction of marijuana use at age 14. Addiction. (2006) 101:1313–22. 10.1111/j.1360-0443.2006.01523.x 16911731

[99] Wang GS. Pediatric concerns due to expanded cannabis use: unintended consequences of legalization. J Med Toxicol. (2017) 13:99–105. 10.1007/s13181-016-0552-x 27139708 PMC5330955

[100] Perez-Reyes M Wall ME. Presence of delta9-tetrahydrocannabinol in human milk. N Engl J Med. (1982) 307:819–20. 10.1056/NEJM198209233071311 6287261

[101] National Library of Medicine (US) Drugs and Lactation Database (LactMed): Cannabis. Bethesda, MD (2019). Available online at: https://www.ncbi.nlm.nih.gov/books/NBK501587/

[102] Cone EJ Bigelow GE Herrmann ES Mitchell JM LoDico C Flegel R. Nonsmoker exposure to secondhand cannabis smoke. III. oral fluid and blood drug concentrations and corresponding subjective effects. J Anal Toxicol. (2015) 39:497–509. 10.1093/jat/bkv070 26139312 PMC4627404

[103] Bergeria CL Heil SH. Surveying lactation professionals regarding marijuana use and breastfeeding. Breastfeed Med. (2015) 10:377–80. 10.1089/bfm.2015.0051 26252053 PMC4692106

[104] Garry A Rigourd V Amirouche A Fauroux V Aubry S Serreau R. Cannabis and breastfeeding. J Toxicol. (2009) 2009:596149. 10.1155/2009/596149 20130780 PMC2809366

[105] Tennes K Avitable N Blackard C Boyles C Hassoun B Holmes L. Marijuana: prenatal and postnatal exposure in the human. NIDA Res Monogr. (1985) 59:48–60. 10.1037/e496932006-005 3929132

[106] Astley SJ Little RE. Maternal marijuana use during lactation and infant development at one year. Neurotoxicol Teratol. (1990) 12:161–8. 10.1016/0892-0362(90)90129-Z 2333069

[107] Liston J. Breastfeeding and the use of recreational drugs–alcohol, caffeine, nicotine and marijuana. Breastfeed Rev. (1998) 6:27–30. 9849117

[108] Holland CL Nkumsah MA Morrison P Tarr JA Rubio D Rodriguez KL. “Anything above marijuana takes priority”: obstetric providers' attitudes and counseling strategies regarding perinatal marijuana use. Patient Educ Counsel. (2016) 99:1446–51. 10.1016/j.pec.2016.06.003 27316326 PMC5007170

[109] Litt MD Kadden RM Petry NM. Behavioral treatment for marijuana dependence: randomized trial of contingency management and self-efficacy enhancement. Addict Behav. (2013) 38:1764–75. 10.1016/j.addbeh.2012.08.011 23254227 PMC3558664

[110] Litt MD Kadden RM Kabela-Cormier E Petry NM. Coping skills training and contingency management treatments for marijuana dependence: exploring mechanisms of behavior change. Addiction. (2008) 103:638–48. 10.1111/j.1360-0443.2008.02137.x 18339108 PMC2697818

[111] Pascale A Consumo de drogas durante el embarazo: efectos sobre el binomio materno-fetal, recién nacido y primera infancia. Modalidades terapéuticas y estrategias de prevención. in: P.N.d.S.d.l.MSP N, (Ed.), Montevideo (2010).

[112] Greenfield L Burgdorf K Chen X Porowski A Roberts T Herrell J. Effectiveness of long-term residential substance abuse treatment for women: findings from three national studies. Am J Drug Alcohol Abuse. (2004) 30:537–50. 10.1081/ADA-200032290 15540492

[113] Conners NA Grant A Crone CC Whiteside-Mansell L. Substance abuse treatment for mothers: treatment outcomes and the impact of length of stay. J Subst Abuse Treatment. (2006) 31:447–56. 10.1016/j.jsat.2006.06.001 17084800

[114] Ashley OS Marsden ME Brady TM. Effectiveness of substance abuse treatment programming for women: a review. Am J Drug Alcohol Abuse. (2003) 29:19–53. 10.1081/ADA-120018838 12731680

[115] Velez ML Jansson LM Montoya ID Schweitzer W Golden A Svikis D. Parenting knowledge among substance abusing women in treatment. J. Subst. Abuse Treat. (2004) 27:215–22. 10.1016/j.jsat.2004.07.004 15501374

[116] Kumpfer KL Fowler MA. Parenting skills and family support programs for drug-abusing mothers. Semin Fetal Neonatal Med. (2007) 12:134–42. 10.1016/j.siny.2007.01.003 17327147

[117] Lester BM Twomey JE. Treatment of substance abuse during pregnancy. Womens Health. (2008) 4:67–77. 10.2217/17455057.4.1.67 19072452

[118] Bara A Manduca A Bernabeu A Borsoi M Serviado M Lassalle O. Sex-dependent effects of *in utero* cannabinoid exposure on cortical function. Elife. (2018) 7:e36234. 10.7554/eLife.36234 30201092 PMC6162091

[119] Shabani M Hosseinmardi N Haghani M Shaibani V Janahmadi M. Maternal exposure to the CB1 cannabinoid agonist WIN 55212-2 produces robust changes in motor function and intrinsic electrophysiological properties of cerebellar Purkinje neurons in rat offspring. Neuroscience. (2011) 172:139–52. 10.1016/j.neuroscience.2010.10.031 20969930

[120] Beggiato S Borelli AC Tomasini MC Morgano L Antonelli T Tanganelli S. Long-lasting alterations of hippocampal GABAergic neurotransmission in adult rats following perinatal Delta(9)-THC exposure. Neurobiol Learn Mem. (2017) 139:135–43. 10.1016/j.nlm.2016.12.023 28104530

[121] Castaldo P Magi S Cataldi M Arcangeli S Lariccia V Nasti AA. Altered regulation of glutamate release and decreased functional activity and expression of GLT1 and GLAST glutamate transporters in the hippocampus of adolescent rats perinatally exposed to Delta(9)-THC. Pharmacol Res. (2010) 61:334–41. 10.1016/j.phrs.2009.11.008 19941959

[122] Antonelli T Tomasini MC Tattoli M Cassano T Tanganelli S Finetti S. Prenatal exposure to the CB1 receptor agonist WIN 55,212-2 causes learning disruption associated with impaired cortical NMDA receptor function and emotional reactivity changes in rat offspring. Cereb Cortex. (2005) 15:2013–20. 10.1093/cercor/bhi076 15788701

[123] Trezza V Campolongo P Cassano T Macheda T Dipasquale P Carratu MR. Effects of perinatal exposure to delta-9-tetrahydrocannabinol on the emotional reactivity of the offspring: a longitudinal behavioral study in Wistar rats. Psychopharmacology. (2008) 198:529–37. 10.1007/s00213-008-1162-3 18452035

[124] Vela G Martin S Garcia-Gil L Crespo JA Ruiz-Gayo M Fernandez-Ruiz JJ. Maternal exposure to delta9-tetrahydrocannabinol facilitates morphine self-administration behavior and changes regional binding to central mu opioid receptors in adult offspring female rats. Brain Res. (1998) 807:101–9. 10.1016/S0006-8993(98)00766-5 9757010

[125] Rodriguez de Fonseca F Cebeira M Fernandez-Ruiz JJ Navarro M Ramos JA. Effects of pre- and perinatal exposure to hashish extracts on the ontogeny of brain dopaminergic neurons. Neuroscience. (1991) 43:713–23. 10.1016/0306-4522(91)90329-M 1922791

[126] Rodriguez de Fonseca F Hernandez ML de Miguel R Fernandez-Ruiz JJ Ramos JA. Early changes in the development of dopaminergic neurotransmission after maternal exposure to cannabinoids. Pharmacol Biochem Behav. (1992) 41:469–74. 10.1016/0091-3057(92)90359-N 1350099

[127] Perez-Rosado A Manzanares J Fernandez-Ruiz J Ramos JA. Prenatal Delta(9)-tetrahydrocannabinol exposure modifies proenkephalin gene expression in the fetal rat brain: sex-dependent differences. Brain Res Dev Brain Res. (2000) 120:77–81. 10.1016/S0165-3806(99)00170-4 10727732

[128] Bonnin de Miguel R Castro JG Ramos JA Fernandez-Ruiz JJ. Effects of perinatal exposure to delta 9-tetrahydrocannabinol on the fetal and early postnatal development of tyrosine hydroxylase-containing neurons in rat brain. J Mol Neurosci. (1996) 7:291–308. 10.1007/BF02737066 8968950

[129] Bayer SA Altman J Russo RJ Zhang X. Timetables of neurogenesis in the human brain based on experimentally determined patterns in the rat. Neurotoxicology. (1993) 14:83–144. 8361683

[130] Clancy B Charvet CJ Darlington RB Finlay BL Workman A Uchiyama R. Translating time across developing mammalian brains. Neuroscience. (2001) 105:7–17. 10.1016/S0306-4522(01)00171-3 11483296

[131] Azzam SM Nielsen MK Dickerson GE. Postnatal litter size effects on growth and reproduction in rats. J Anim Sci. (1984) 58:1337–42. 10.2527/jas1984.5861337x 6540258

[132] Agnish ND Keller KA. The rationale for culling of rodent litters. Fundam Appl Toxicol. (1997) 38:2–6. 10.1093/toxsci/38.1.2 9268601

